# OsALB3 Is Required for Chloroplast Development by Promoting the Accumulation of Light-Harvesting Chlorophyll-Binding Proteins in Rice

**DOI:** 10.3390/plants12234003

**Published:** 2023-11-28

**Authors:** Chao Zhang, Xinchen Mao, Xiaoxiao Feng, Yali Sun, Zirui Wang, Jiaqi Tang, Hengxiu Yu

**Affiliations:** 1 Jiangsu Key Laboratory of Crop Genomics and Molecular Breeding/Key Laboratory of Plant Functional Genomics of the Ministry of Education/Jiangsu Key Laboratory of Crop Genetics and Physiology, Agricultural College of Yangzhou University, Yangzhou 225009, China; chaozhang@yzu.edu.cn (C.Z.); 13584220802@163.com (X.M.); 13952758764@163.com (X.F.); mz120201234@stu.yzu.edu.cn (Y.S.); 1113544933@163.com (Z.W.); tjq990821@163.com (J.T.); 2 Jiangsu Co-Innovation Center for Modern Production Technology of Grain Crops, Yangzhou University, Yangzhou 225009, China

**Keywords:** OsALB3, chloroplast development, LHCPs, thylakoid membrane, rice

## Abstract

ALBINO3 (ALB3) protein functions in the insertion and assembly of thylakoid membrane protein complexes and plays a critical role for chloroplast development in *Arabidopsis*. However, the biological function of ALB3 homologs in rice, OsALB3, remains elusive. Here, we identified a rice mutant, *yellow leaf and lethal1* (*yll1*), that displayed yellow leaves and died at the seedling stage. The content of chlorophyll in *yll1*, compared with wild type, was significantly decreased. Transmission electron microscopy observation shows that the chloroplast of *yll1* lacks thylakoid membranes. The causal mutation, which is located in *OsALB3*, was isolated by Mutmap+ combined with a simple mutation filtering process. Knockout of *OsALB3* leads to yellow leaves and seedling lethality, mimicking the phenotype of *yll1*. *OsALB3* is widely expressed and OsALB3 is chloroplast-localized. Moreover, the content of light-harvesting chlorophyll-binding proteins in *yll1* is reduced. Together, our study demonstrated the essential role of OsALB3 in chloroplast development and provided clues to the possible conserved molecular function of ALB3 in rice.

## 1. Introduction

Chloroplasts are semi-autonomous organelles that contain DNA and gene expression apparatus [1]. However, the majority of proteins in chloroplasts are encoded in the nucleus and transferred post-translationally into the chloroplasts [2,3]. Thylakoid-located proteins are further transported from chloroplast stroma into the thylakoid membranes or lumen through at least four different pathways [4]. Among these pathways, the signal recognition particle (cpSRP) pathway facilitates targeting of the light-harvesting chlorophyll a/b-binding proteins (LHCPs) and involves the chloroplast signal recognition particle, cpSRP54 and cpSRP43, as well as its membrane receptor FtsY and the translocase ALB3 [5,6]. Briefly, the imported LHCPs bind to cpSRP43 [7], and cpSRP54 interacts with cpSRP43 to form a soluble transit-complex [8]. Then, receptor protein cpFtsY, together with ALB3, facilitates the insertion of the complex into the thylakoid membrane [9]. Functional disruption of the participant in the signal recognition particle pathway leads to chlorotic leaves [10,11,12,13,14,15,16].

*albino3* (*alb3*), a mutant with colorless cotyledons, was first identified in *Arabidopsis* through Ac/Ds mutagen system [17]. Detailed characterization showed that *alb3* contains significantly less chlorophyll than wild type. The chloroplast morphology of *alb3* is less organized, with few thylakoid membranes, barely any grana stacking, and no starch grains. Interestingly, ALB3 shows similarity to a bacterial membrane protein YidC, a component of the bacterial translocation machinery, and to yeast mitochondrial protein OXA1, a translocase component used to integrate a subset of inner membrane proteins encoded by both mitochondrial and nuclear genomes [18,19,20]. Similarly, ALB3 is required for integration of the LHCPs, which are exclusively encoded in the nucleus, into thylakoid membranes [5,21]. ALB3 also seems to be involved in cotranslational assembly of certain chloroplast-encoded membrane proteins, such as members of cytochrome b_6_ complex, by interacting with ribosomes [22,23]. Similarly, depletion of *ALB3.1* in *Chlamydomonas reinhardtii* results in a clear impairment in LHCP accumulation and a diminished amount of photosystem II [21,24]. Loss of ALB3b results in truncated light-harvesting antenna in diatoms [25].

As an important insertase in chloroplast, ALB3 was proved to interact with multiple proteins and complexes. ALB3 is able to form dimers or oligomers. ALB3 interacts directly with thylakoid membrane proteins cpSecE and cpSecY, two members of the cpSec-translocase. ALB3 was also shown to bind to the PSII proteins, to the PSI reaction center protein PsaA, and the ATP synthase subunit CF_0_III, suggesting its important role in the assembly of photosynthetic thylakoid membrane complexes [26]. The C-terminal domain of ALB3 interacts with cpSRP43, a stromal cpSRP component, and is also important for the stability of ALB3 in a light dependent manner [27,28,29,30]. Also, ALB3 interacts with *Arabidopsis* Tellurite resistance C protein (AtTerC) to facilitate de novo synthesis of thylakoid membrane proteins at the membrane insertion step [31].

Despite the extensively studied function of ALB3 in dicotyledon and lower plants, the biological functions of rice ALB3 remain elusive. In this study, we cloned the *OsALB3* gene through *yellow leaf and lethal1* (*yll1*) mutant and demonstrated that OsALB3 is required for chloroplast development by promoting the accumulation of LHCPs.

## 2. Results

### 2.1. Characterization of Yellow Leaf and Lethal 1 Mutant

**Figure 1:**
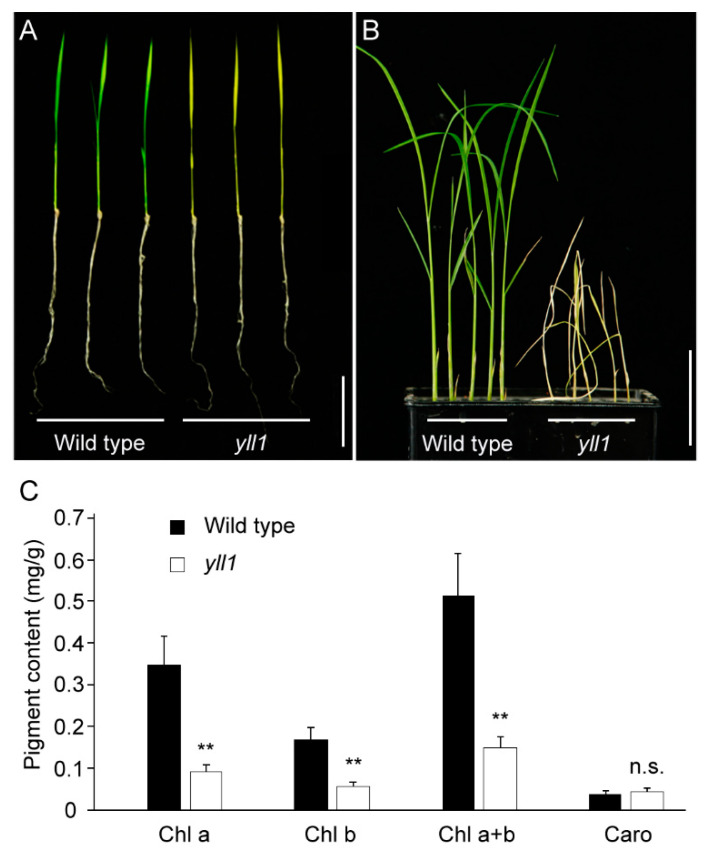
Phenotypic characterization of *yll1.* (**A**) Comparison of the ten-day-old seedlings of wild type and *yll1*. Scale bar = 5 cm. (**B**) Comparison of the three-week-old seedlings of wild type and *yll1*. *yll1* died at this time point. Scale bar = 5 cm. (**C**) Photosynthesis pigment content in two-week-old seedlings of wild type and *yll1*. Asterisks indicate significant differences according to two-tailed Student’s *t* test (** *p* < 0.01). Chl, Chlorophyll. Caro, Carotenoid. n.s., no significance.

Through mutagenesis, we obtained a rice mutant with changed leaf color. This mutant exhibited yellow leaves from the emergence of the first leaf and died within three weeks (Figure 1A,B). The phenotype of this mutant was steadily inherited for several generations and we named this mutant *yellow leaf and lethal 1* (*yll1*).

We compared the photosynthesis pigment content of two-week-old wild type and *yll1* seedlings. The *yll1* contains significantly less Chl a (0.35 ± 0.07 mg/g in wild type and 0.093 ± 0.02 mg/g in *yll1*) and Chl b (0.17 ± 0.03 mg/g in wild type and 0.057 ± 0.01 mg/g in *yll1*) than that of wild type. The content of carotenoid between wild type and *yll1* is comparable (Figure 1C).

### 2.2. Chloroplast Development Was Impaired in yll1

**Figure 2:**
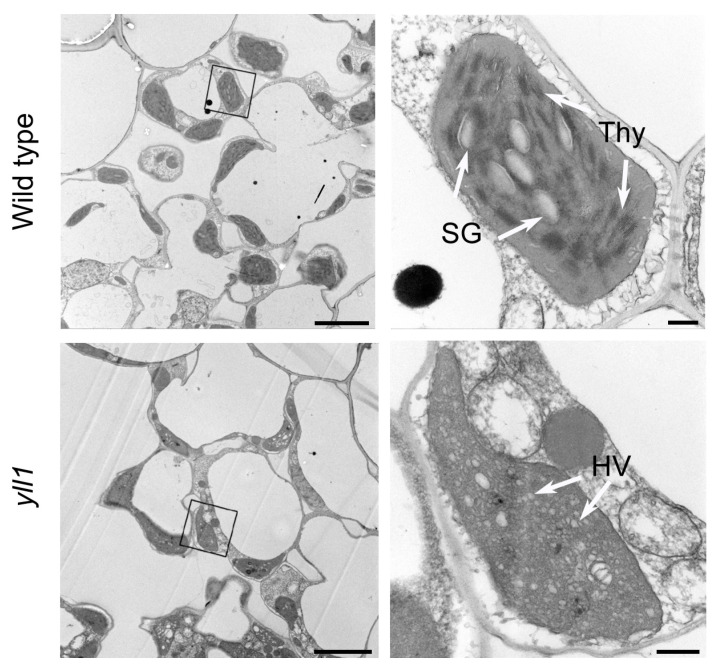
Transmission electron microscopy of leaves of wild type and *yll1* plants. The second leaves of the ten-day-old seedlings of wild type and *yll1* were used. Right panels are the magnification of the black box in respective left panels. Thy, thylakoid. HV, hollow vesicles. SG, starch granule. Scale bars = 5 μm in left panels, 0.5 μm in right panels.

To investigate the chloroplast development in *yll1*, we compared the ultrastructure of chloroplasts in leaves from wild type and *yll1* using transmission electron microscopy (TEM) (Figure 2). In wild type, fully developed chloroplasts with starch granules and massive thylakoid membranes were observed. In contrast, chloroplast morphology of *yll1* seems less organized. Notably, few thylakoid membranes are present in *yll1* chloroplast. The number of stacks of grana per chloroplast in *yll1* (1.86 ± 1.21, n = 7) is significantly fewer than that of wild type (22.43 ± 7.98, n = 7) (Figure S1). Instead, a large number of hollow vesicles were observed in the chloroplast of *yll1*. This indicated that the development of chloroplast is impaired in *yll1*. The chloroplast sizes of wild type and *yll1* are comparable (Figure S2).

### 2.3. Identification of the Causal Mutation through Mutmap+

The phenotype of *yll1* is consistent in descendants of heterozygote for several generations. M5 progenies of M4 plants showed a 3:1 (102:30, χ^2^(1) = 0.36, *p* > 0.05) segregation ratio between wild type phenotype plant and mutant, indicating that the mutant phenotype is caused by a single recessive nuclear gene mutation.

The Mutmap+ approach, a method based on high throughput sequencing, was used to isolate the causal gene [32]. The genomic DNA of 30 wild type progenies and 30 mutant-type progenies of the M4 plant mentioned above were mixed in equal proportion to produce two DNA bulks. The wild type bulk and mutant bulk were separately sequenced to generate Illumina HiSeq paired-end reads that were mapped to reference genomes of *Nippobare* (http://rice.uga.edu (accessed on 7 December 2021)). The average sequence depth for wild type bulk and mutant bulk is 18.06× and 20.90×, respectively. SNP/Indel-index was calculated as the ratio between the number of reads of a mutant SNP/Indel and the total number of reads corresponding to the SNP/Indel. After the removal of SNP/Indel with SNP/Indel-index < 0.3 in both bulks, 22,509 SNPs and 4250 Indels, compared to the reference genome, were identified (Table S1).

**Figure 3:**
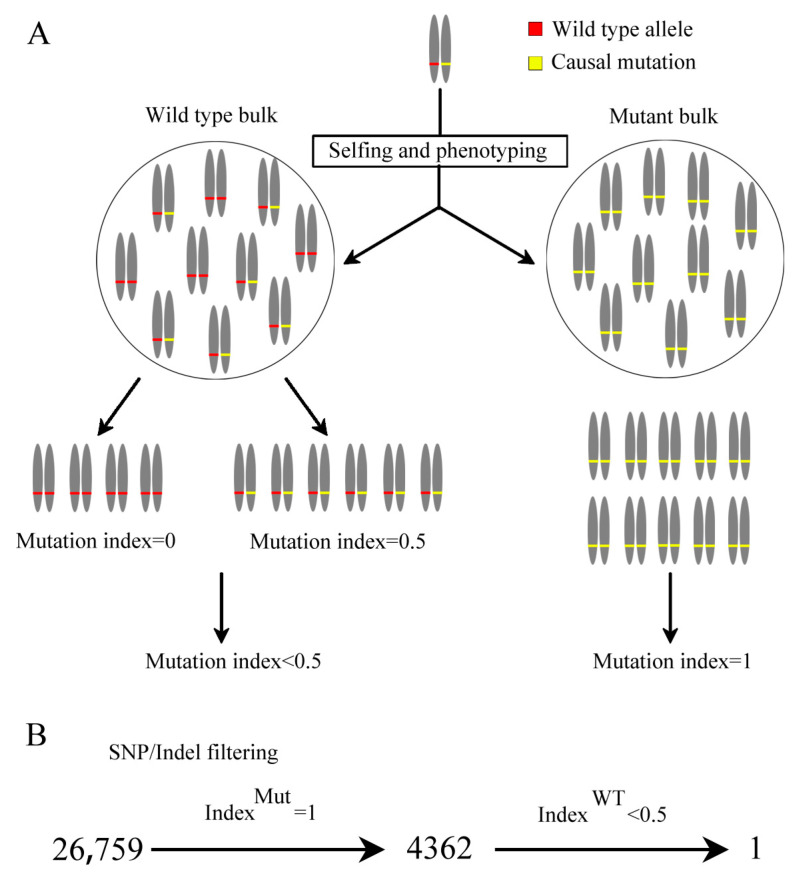
Isolation of causal mutation through Mutmap+ and mutation filtering process. (**A**) Diagram for the mutation filtering criteria for Mutmap+. Grey bars symbolize homologous chromosomes. Red and yellow labels indicate wild type allele and causal mutations, respectively. (**B**) Mutation filtering process for *yll1*. Numbers indicate the quantity of SNP/Indel after each filtering. Filter parameters are indicated above the arrows.

Based on the principles of genetics, we set criteria to isolate the causal mutation for the phenotype of *yll1* (Figure 3A). They are: (1) SNP/Indel-index of mutant bulk is 1 as the phenotype of *yll1* is unambiguous. (2) SNP/Indel-index of wild type bulk is <0.5 as it contains wild type, with SNP/Indel-index = 0, and heterozygotes, with expected SNP/Indel-index = 0.5. These criteria are not strictly rigorous, as sequencing error and biases are neglected. After this filtration, only one SNP, with SNP-index = 1 (29 mutation depth/29 total depth) and 0.12 (2 mutation depth/16 total depth) in mutant bulk and wild type bulk respectively, was identified (Figure 3B). This SNP represents a substitution from C to T in 2,771,134 bp of chromosome 1.

To verify the results of Mutmap+ and our mutation filtration process, we sequenced 20 randomly selected seedlings from segregating populations using Sanger sequencing. As expected, phenotype co-segregated with genotype. All mutants presented homologous mutation of the candidates’ SNP, and seedlings without mutant phenotype were a combination of wild type sequence and heterozygote (Figure S3).

### 2.4. Characterization of OsALB3

**Figure 4:**
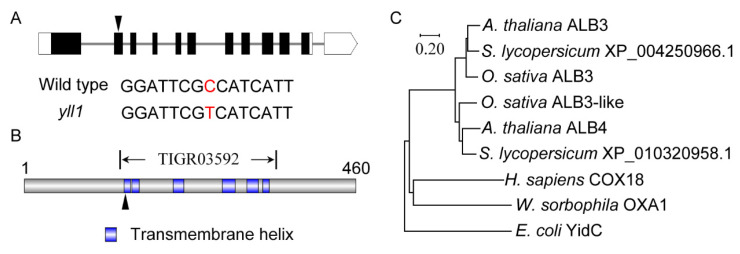
Characterization of OsALB3: (**A**) Gene structure of *OsALB3*. Coding regions are shown as black boxes. The 5′- and 3′-untranslated regions are shown as white boxes. Introns are shown as grey lines. The triangle indicates the position of mutation in *yll1*. Details of sequence modification in *yll1* are listed below. Mutated nucleotide in *yll1* is in red. (**B**) Schematic representation of the conserved transmembrane helix domains in OsALB3. The triangle indicates the position of amino acid change in *yll1*. (**C**) Phylogenetic tree derived from the full-length amino acid sequences of OsALB3 homologs in different species. *A. thaliana*, *Arabidopsis thaliana*. *S. lycopersicum*, *Solanum lycopersicum*. *O. sativa*, *Oryza sativa*. *H. sapiens*, *Homo sapiens*. *W. sorbophila*, *Wickerhamiella sorbophila*. *E. coli*, *Escherichia coli*. The tree was constructed using Mega X based on the neighbor-joining method.

According to the Rice Genome Annotation Project, the candidate SNP, 2,771,134^th^ bp of chromosome 1, located at the second exon of gene *LOC_Os01g05800*. *LOC_Os01g05800,* is annotated as the coding gene of an inner membrane protein which shows similarity (73% identities and 83% positives) with *Arabidopsis* ALBINO3 (ALB3). We thus designated this gene *OsALB3*. *OsALB3* consists of 12 exons (Figure 4A). The open reading frame (ORF) is 1383 bp in length, and the deduced protein contains 460 amino acids. Mutation of *OsALB3* in *yll1* substitutes Ala^140^ of OsALB3 by Val^140^.

OsALB3 contains a YidC_Oxa1_C term (accession: TIGR03592) domain in the central part (Figure 4B). Six transmembrane helixes were predicted in this domain. The first and the second transmembrane helixes are separated by only one lysine residue. Additionally, we executed BLAST searches to identify homologs of OsALB3 in other species. The results showed that homologs of OsALB3 are present in plants, animals, fungus, and bacterium. Moreover, besides ALB3, additional ALB3-like proteins were identified in plant species (*Oryza sativa*, XP_015631158.1, encoded by LOC_Os03g62750; *Arabidopsis thaliana*, NP_173858.5, encoded by At1g24490; *Solanum Lycopersicum*, XP_010320958.1, encoded by LOC101252687). Phylogenetic analysis showed that plant ALB3 homologs are more closely related, and that ALB3 and ALB3-like proteins (ALB4 in *Arabidopsis*) constitute isolated branches in the phylogenetic tree (Figure 4C). Multiple sequence alignment revealed that ALB3 and ALB3-like proteins are highly conserved within the YidC_Oxa1_C term domain (Figure S4). The mutated amino acid in *yll1* resides in the first transmembrane helix of OsALB3 (Figure 4B) and is conserved among different species (Figure S4).

### 2.5. Knockout of OsALB3 Mimics the Phenotype of yll1

**Figure 5:**
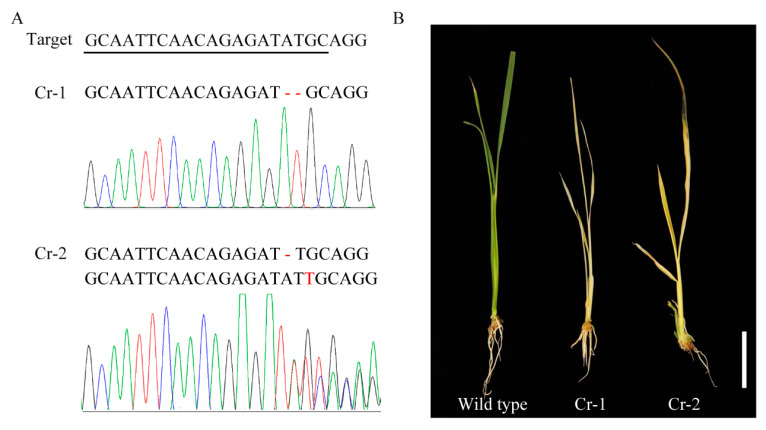
Knockout of *OsALB3* leads to *yll1*-like phenotype. (**A**) Target sequence for CRISPR-Cas9 gene editing and genotype of knockout mutants (Cr-1 and Cr-2) of *OsALB3*. Mutated nucleotides in mutants are in red. (**B**) Phenotypes of knockout mutants of *OsALB3* at four-leaf stage. Scale bar = 5 cm.

To confirm the biological function of *OsALB3*, we used the CRISPR/Cas9 gene editing approach to knock out this gene in wild type plants. A target sequence located in the third exon was picked to construct the vector. Sequence analysis showed that 2 of 11 T_0_ lines, Cr-1 and Cr-2, harbor putative null alleles of *OsALB3*. There is 2 bp deletion at the target site in Cr-1. In Cr-2, the genotype is constituted of 1 bp deletion and 1 bp insertion, both of which putatively lead to frame shift of *OsALB3* (Figure 5A). Cr-1 and Cr-2 lines displayed yellow leaves and died eventually, mimicking the phenotype of *yll1* (Figure 5B). This confirmed that the mutant phenotype is indeed caused by *OsALB3* dysfunction.

### 2.6. OsALB3 Is Located in Chloroplasts

To examine the expression pattern of *OsALB3*, we carried out qRT-PCR using different rice tissues. The results showed that *OsALB3* transcripts were detected in roots, internodes, leaves, and panicles, with the highest expression level in leaves (Figure S5). The expression levels of *OsALB3* in wild type and *yll1* are comparable (Figure S6).

**Figure 6:**
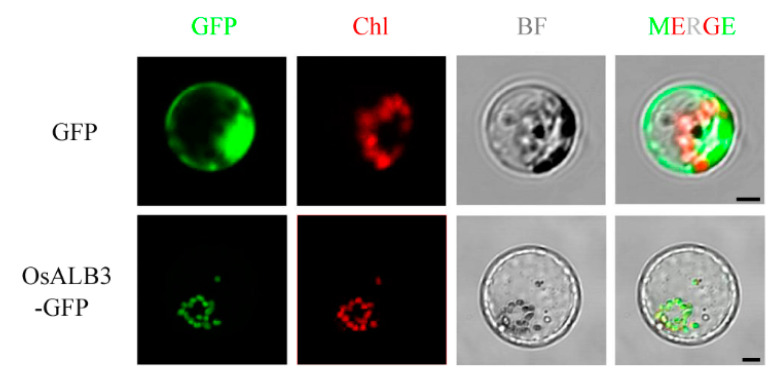
Subcellular localization of OsALB3 in rice protoplasts. Green fluorescence indicates GFP signal, red fluorescence indicates chloroplast autofluorescence. Chl, chloroplast. BF, bright field. Scale bars = 5 μm.

To examine the subcellular localization of OsALB3, we fused OsALB3 with green fluorescent protein (GFP) and performed a transient expression assay in rice protoplasts. The result showed that the green signal of OsALB3-GFP overlapped with the chlorophyll autofluorescence signal, whereas the free GFP signal displayed a ubiquitous distribution pattern throughout the cell (Figure 6). As with OsALB3, OsALB3^Ala140Val^ also overlapped with the chlorophyll autofluorescence (Figure S7). This indicated that OsALB3 is located in chloroplasts, which is consistent with its biological function in chloroplasts’ development.

### 2.7. Light-Harvesting Chlorophyll-Binding Proteins Were Reduced in yll1

**Figure 7:**
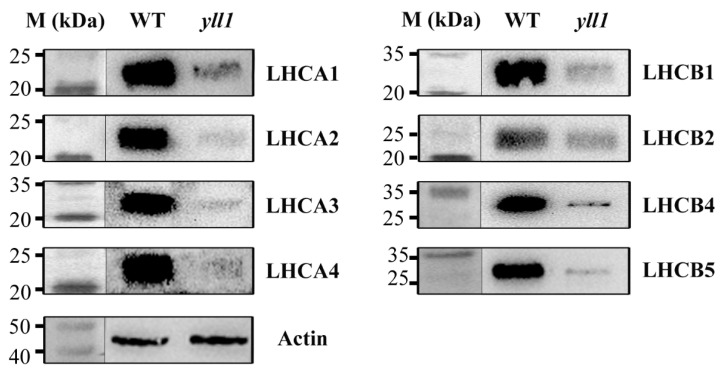
Western blot analysis of light-harvesting chlorophyll-binding proteins from the wild type (WT) and *yll1*. Proteins of wild type and *yll1* were isolated and probed with antisera against LHCA and LHCB proteins. Actin was used as a loading control to normalize the sample amount. Protein markers (M) with labeled sizes are shown, left.

It has been shown that *Arabidopsis* ALB3 is critical for chloroplast development by integrating the light-harvesting chlorophyll-binding proteins (LHCPs) into thylakoid membranes [5]. To test whether OsALB3 participates in LHCP accumulation, we performed immunoblot analysis using antisera directed against LHCA and LHCB to determine their levels. As shown in Figure 7, LHCA2, LHCA3, and LHCA4 were barely detected in *yll1* and the level of LHCA1 in *yll1* was significantly decreased when compared with that of wild type. Similarly, LHCB1, LHCB2, LHCB4, and LHCB5 were also less accumulated in the *yll1* mutant. This indicated the essential role of OsALB3 in LHCP accumulation. We compared the expression level of LHCPs’ encoding genes between wild type and *yll1*. All these genes (*LHCA1*, *LHCA2*, *LHCA3*, *LHCA4*, *LHCB1*, *LHCB2*, *LHCB4*, and *LHCB5*) showed no significant differences (Figure S8). This indicated that the reduced accumulation of LHCPs in *yll1* is not caused by transcription defects of LHCPs’ encoding genes.

## 3. Discussion

Dissecting the molecular mechanisms of chloroplast development in crops might pave the way for breeding with high photosynthetic efficiency and production. In this study, we proved that OsALB3 is required for chloroplast development in rice by characterizing a novel rice chlorophyll-deficient mutant *yll1*. Cytological observation showed that chloroplast of *yll1* lacks thylakoid membranes. Instead, extensive hollow vesicles with whitish staining were observed in the chloroplast of *yll1* (Figure 2). These loosely organized vesicles were also present in the mutant of *OscpSRP54b*, a gene involved in rice chloroplast development [16]. The accurate constitution of these vesicles has been obscured recently. We verified that mutation of *OsALB3* is responsible for the phenotype of *yll1* by Mupmap+ and gene editing approaches (Figure 3 and Figure 5). Mupmap+ is reported to be suitable for identifying mutations that cause early development lethality or sterility, or generally hamper crossing [32]. We proved that accumulation of LHCPs is impaired in *yll1*, which leads to the absence of thylakoid membranes and dysfunctional chloroplast.

The mutation of *OsALB3* in *yll1* leads to substitution from alanine to valine in the 140th amino acid of OsALB3. This amino acid (Ala^140^) resides in the first transmembrane helix of OsALB3 (Figure 4B) and is conserved among different species (Figure S4). Both alanine and valine are nonpolar and hydrophobic. How a single amino acid substitution from Ala^140^ to Val^140^ of OsALB3 in *yll1* changes the protein function is a fascinating question. There are multiple possible underlying mechanisms, including gene expression level, protein localization pattern, interactions with other proteins, and so on. We compared the expression level of *OsALB3* in wild type and *yll1*. The results showed that there is no significant difference between them (Figure S6). The subcellular localization pattern of OsALB3^Ala140Val^ was checked. As with OsALB3, OsALB3^Ala140Val^ was also localized in chloroplast (Figure S7). Thus, how a single amino acid substitution from Ala^140^ to Val^140^ of OsALB3 in *yll1* changes the protein function remains elusive.

Besides ALB3, another ALB3 ortholog is present in plant species [33]. In *Arabidopsis*, this *ALB3*-like gene was named *ALB4* and proved to be required for proper chloroplast biogenesis [34]. Further studies reported that ALB4 functions in the assembly of the CF_1_CF_0_-ATP synthase. Accumulation and stoichiometric assembly of its subunits, which are of dual genetic origin, are highly affected in plants depleted of ALB4 [35]. Genetic and physical interactions between ALB4 and ALB3 suggest that the two ALB proteins may engage similar sets of interactors for their overlapping functions [36]. In *Chlamydomonas reinhardtii*; ALB3.2, the second ALB3 homolog; interacts with ALB3.1, the reaction center polypeptides of PSI and PSII; and VIPP1, a protein involved in thylakoid formation [21]. Rice also contains an ALB3-like protein (XP_015631158.1) which is encoded by *LOC_Os03g62750* (Figure 4C). According to the public database, this gene is also ubiquitously expressed (https://www.ncbi.nlm.nih.gov/gene/4334759 (accessed on 5 May 2023)). The clarification of the biological functions of the ALB3-like protein in rice, and its relationship with OsALB3, require further studies.

The chloroplast protein accumulations are highly associated with chloroplast function. Overexpression of OsTLP27, a thylakoid lumen protein of unknown function in chloroplast, resulted in increased pigment content and enhanced photochemical efficiency [37]. The overexpression of *MdLhcb4.3* in transgenic *Arabidopsis* and apple callus enhanced their tolerance to drought and osmotic stress [38]. During infection by the rice blast fungus *Magnaporthe oryzae*, the *LHCB5*-OX rice lines showed strong resistance, with punctate and significantly reduced lesion areas, whereas the *lhcb5*-RNAi lines were more susceptible [39]. Moreover, in rice, modulation of the expression of Golden2-like factors (GLKs; transcriptional activators of genes encoding LHCPs), boosts rice chloroplast development, photosynthesis, and grain yield [40,41]. Thus, it is tempting to test whether manipulation of *OsALB3* expression level (e.g., over expression) leads to increased LHCP levels and enhanced chloroplast functions.

In *Arabidopsis*, ALB3 was proved to be firmly associated with thylakoid membranes and interact with FtsY, cpSRP43, and AtTerC [9,27,31,42]. *Arabidopsis cpftsY* mutant plants had yellow leaves in which the levels of LHCPs were reduced to 10–33% compared with wild type. In contrast, *alb3* had yellowish white leaves, and the LHCP levels were less than or equal to 10% of those of wild type. This indicated that these two membrane-bound cpSRP components contribute non-identically to thylakoid biogenesis [9]. AtTerC is nuclear-encoded, and shares sequence similarity with bacterial TerC, which is the product of a member of an operon associated with tellurite resistance. Mutation of *AtTerC* leads to reduced levels of PS II core proteins and pale-green cotyledons [31]. These interactions between OsALB3 and players in signal recognition particle pathways, and the biological significances of putative interactions, remain to be elucidated.

## 4. Materials and Methods

### 4.1. Plant Materials

The *yll1* mutant was isolated from a collection of mutants of our lab, which were induced by ethane methyl sulfonate (EMS) on *japonica* rice (*Oryza sativa*) variety, Nipponbare. Heterozygous plants were cultivated for several generations to confirm the stability of the mutant phenotype. Genotyping on *OsALB3* of rice individuals was performed by PCR and Sanger sequencing. The variety Nipponbare was used as the wild type in all experiments. Plant materials were cultivated in experimental plot and incubator with regular water and fertilizer management. The photoperiod and temperature of incubator was 14 h/30 °C in light, with 20,000 Lux, and 10 h/28 °C in dark.

### 4.2. Measurement of Pigment Content

Pigment contents were determined by the previous method with minor modifications [43]. A quantity of 0.1 g fresh leaves was cut and soaked in a 10 mL solution of 1:1 ethanol/acetone (*v*/*v*). After 12 h of treatment in darkness, the absorbance values at 470, 649, and 665 nm wavelength were measured using a Microplate Reader. The pigment contents were calculated based on the absorbance values. The calculation formula is as follows: Chl a = (12.21 × OD_665_ − 2.81 × OD_649_) × V/W; Chl b = (20.13 × OD_649_ − 5.03 × OD_665_) × V/W; Caro = (1000 × OD_470_ × V/W − 3.27 × Chl a – 104 × Chl b) /198. All experiments were carried out with three biological replicates.

### 4.3. Transmission Electron Microscopy Analysis

Fresh leaves (the second leaves at the ten-day-old seedling of wild type and *yll1*) were cut into pieces and fixed in 2.5% glutaraldehyde in a phosphate buffer for 4 h. After washing by phosphate buffer (0.1 M) three times, samples were fixed in 1% OsO_4_ for 1.5 h. After the fixation, phosphate buffer (0.1 M) was used again to wash the samples three times. Samples were dehydrated through an acetone series (50% for 15 min, 70% for 15 min, 90% for 15 min, 100% for 20 min, 20 min, and 20 min) and finally embedded in Epon812 resin (acetone: embedding = 2:1 for 0.5 h, acetone: embedding fluid = 1:2 for 1.5 h at 37 °C, embedding fluid for 3 h at 37 °C). After solidifying in 37 °C, 45 °C, 60 °C for 24 h, respectively, ultrathin sections (70 nm) were produced using Reichert-Jung ULTRACUT E (Vienna, Austria) slicer. Samples were double stained with uranyl acetate and lead citrate for 15 min, respectively. Sections were observed with a JEM 1200 transmission electron microscope.

### 4.4. Mutmap+

The rice line with phenotype segregation was screened and leaves of seedlings with and without mutant phenotype were harvested, respectively. Extracted DNA from individual plants of two populations was mixed in equal proportion, according to concentration, to produce DNA pools. High-through sequencing and data analysis were performed by Novogene Co., Ltd. Briefly, qualified DNA samples were randomly broken into fragments with 350 bp using a Covaris crusher. DNA fragments were processed through terminal repair, addition of ploy A tail, addition of sequencing adaptors, purification, PCR amplification, and other steps to construct the library (TruSeq Library Construction Kit). Illumina HiSeq™ PE150 assay was used to sequence the library. After quality control of raw data and genome mapping, SNP/Indel were detected and annotated by GATK3.8 [44]. All SNP/Indel detected from two libraries were listed in Table S1. SNP/Indel were filtered based on the process depicted in Figure 3.

### 4.5. Knockout of OsALB3 by CRISPR/Cas9 Gene Editing Approach

The CRISPR/Cas9 gene editing approach in rice was performed according to a protocol described previously [45]. Briefly, target sequence was designed using CRISPR-GE, a convenient software toolkit for CRISPR-based genome editing (http://skl.scau.edu.cn/ (accessed on 1 August 2023)). OsU6a promoter, target sequence, and gRNA were linked through two rounds of PCR. In the first round PCR, target sequence was linked to OsU6a promoter and gRNA, respectively, through designed primers. In the second round PCR, mixed DNA from the first PCR, both of which harbored the overlapped target sequence, was used as template to link three elements. The PCR products were cloned into pYLCRISPR/Cas9-MH vector by *Bsa* I digestion and T4 ligation [45]. The resulting constructs were sequenced and transformed into *Agrobacterium tumefaciens* EHA105 and then into calli of *Nipponbare*, a japonica variety. T_0_ plants were genotyped to verify whether the editing site was modified. Photos of wild type and *yll1* seedlings were taken at four-leaf stage.

### 4.6. qRT-PCR Assay

RNA from different tissues was extracted by Trizol reagent. Reverse transcript PCR was performed using HiScript II Q RT SuperMix (+gDNA wiper) (Vazyme, Nanjing, China). Genomic DNA was removed by gDNA wiper in the reverse transcript PCR mix. Oligo (dT)_23_ primer was used to trigger the PCR reaction. Real-time PCR analysis was performed using the Bio-Rad CFX96 real-time PCR instrument and ChamQ SYBR qPCR Master Mix (Vazyme). All PCR experiments were conducted using 40 cycles of 95 °C for 10 s, 60 °C for 30 s. All reactions were performed in triplicate, with *Ubiqutin* as the normalized reference gene for all comparisons. ΔΔCt method was used to calculate the relative gene expression. Quantification of the expression level of LHCPs’ encoding genes followed the same procedure. The primers for qRT-PCR are listed in Table S2.

### 4.7. Subcellular Localization of OsALB3

The coding sequence of *OsALB3* was amplified and cloned into pJIT163-GFP vector (Biovector, Beijing, China) in frame with GFP. Plasmids were transfected into rice protoplasts through PEG-mediated transformation. Empty pJIT163-GFP vector was used as the control. Fluorescence signals were captured at 24 h after transformation with a confocal laser scanning microscope (Carl Zeiss LSM 710, Oberkochen, Germany). GFP and red fluorescence signals (chloroplast auto-fluorescence) were excited at 488 and 561 nm, and emissions were collected at 500–540 nm and 600–650 nm, respectively. The primers for PCR are listed in Table S2.

### 4.8. Western Blot Analysis

Western blotting was performed as previously described [46]. Proteins were extracted from young rice seedlings with extract buffer solution (10 mM Tris-HCl pH 7.5, 2 mM EDTA, 250 mM HCl, 5 mM DTT, and protease inhibitor). Protein samples were separated by SDS-PAGE on a 12% polyacrylamide gel and electroblotted onto PVDF (polyvinylidene difluoride) membranes. Western blots were performed with primary antibodies diluted 1:1000 and goat anti-rabbit IgG antibodies conjugated to horseradish peroxidase diluted 1:10,000. Actin was used as loading control in the western blot assay to normalize the sample amount.

### 4.9. Sequence Alignment and Analysis

Transmembrane prediction was performed by DeepTMHMM (Version 1.0.24) (https://dtu.biolib.com/DeepTMHMM (accessed on 13 June 2023)). The phylogenetic tree was constructed using MEGA version X based on the neighbor-joining method. Poisson models and uniform rate categories were used in distance analyses. The complete deletion option was applied. Multiple alignments were conducted using MAFFT (https://toolkit.tuebingen.mpg.de/mafft (accessed on 21 March 2023)) with 1.53 gap open penalty and 0 offset. The alignment was processed with ESPrint (http://espript.ibcp.fr/ESPript/ESPript/ (accessed on 1 August 2023)) with the equivalent coloring scheme. Default global score 0.7 was used to define the threshold between low and high similarity [47].

## 5. Conclusions

OsALB3 is a chloroplast-localized protein with transmembrane helix domains. OsALB3 promotes the accumulations of LHCPs to maintain normal chloroplast development. Mutation of *OsALB3* leads to yellow leaves and seedling lethality of rice.

## Data Availability

All of the raw data of genomic resequencing in this research have been deposited in the Sequence Read Archive (SRA) database of NCBI (SRR24966869, SRR24966870) in BioProject accession PRJNA985070.

## Supplementary Materials

The following supporting information can be downloaded at: https://www.mdpi.com/article/10.3390/plants12234003/s1, Figure S1: Comparison of the number of thylakoids per chloroplast between wild type and *yll1*. Asterisks indicate significant differences according to two-tailed Student’s *t* test (** *p* < 0.01); Figure S2: Comparison of the chloroplast size between wild type and *yll1*. Error bars represent SD. n.s. indicates no significant differences according to two-tailed Student’s *t* test (*p* = 0.86); Figure S3: Genotype of the causal mutation in plants with different phenotypes. Red arrow indicates the 2,771,134th of chromosome 1; Figure S4: Multiple sequence alignment of the ALB3 and ALB3-like proteins in *Oryza sativa*, *Arabidopsis thaliana*, and *Solanum Lycopersicum*. The conserved YidC_Oxa1_Cterm domain is underlined in blue; Figure S5: Real-time PCR analysis of *OsALB3* in different rice tissues. *Ubiquitin* is used as endogenous control. Error bars represent SD (n = 3); Figure S6: Comparison of expression levels of *OsALB3* between wild type and *yll1*. Seedling leaves were used for real-time PCR analysis. *Ubiquitin* is used as endogenous control. Error bars represent SD (n = 3). n.s. indicates no significant differences according to two-tailed Student’s *t* test; Figure S7: Subcellular localization of OsALB3^Ala140Val^ in rice protoplasts. Green fluorescence indicates GFP signal, red fluorescence indicates chloroplast autofluorescence. Chl, chloroplast. BF, bright field. Scale bar = 5 μm; Figure S8 Comparison of expression levels of LHCPs’ encoding genes between wild type and *yll1*. Seedling leaves were used for real-time PCR analysis. Ubiquitin is used as endogenous control. Error bars represent SD (n = 3). n.s. indicates no significant differences according to two-tailed Student’s t test. Table S1: Genome-wide SNP index of mutant bulk and wild type bulk, Table S2: Molecular markers used in this study.
