# Supplementary material for: OsALB3 Is Required for Chloroplast Development by Promoting the Accumulation of Light-Harvesting Chlorophyll-Binding Proteins in Rice
Source: Plants (Basel). 2023 Nov 28;12(23):4003. doi: 10.3390/plants12234003 (PMC10707891; doi:10.3390/plants12234003)
Supplement: Supplementary file 1 [file plants-12-04003-s001.zip › Supplymental figures.pdf]

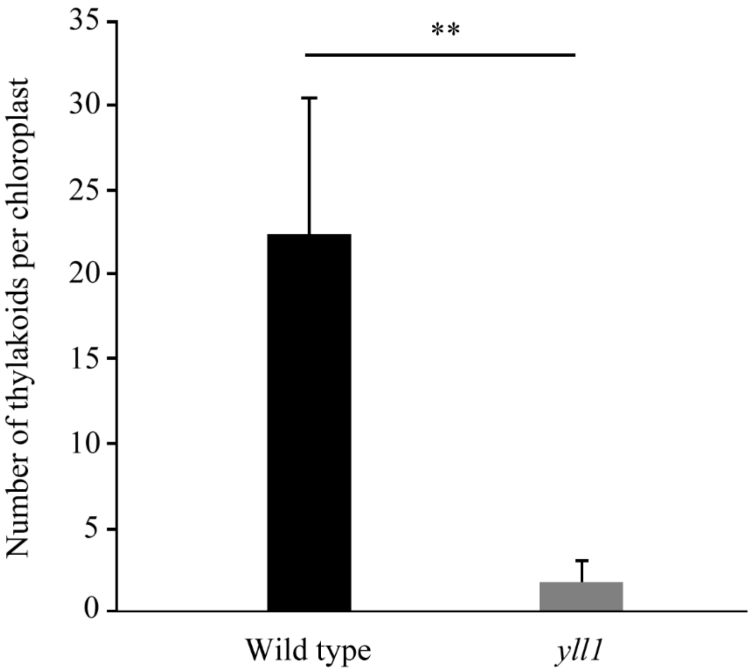


Figure S1 Comparison of the number of thylakoids per chloroplast between wild type and *yll1*. Asterisks indicate significant differences according to two-tailed Student's *t* test (***P* <0.01).

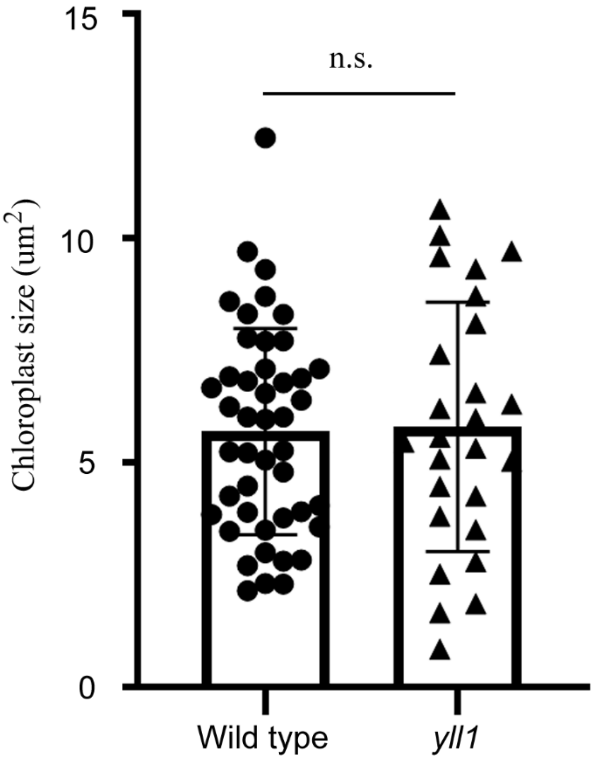


Figure S2 Comparison of the chloroplast size between wild type and *yll1*. Error bars represent SD. n.s. indicate no significant differences according to two-tailed Student's *t* test (*P*=0.86).

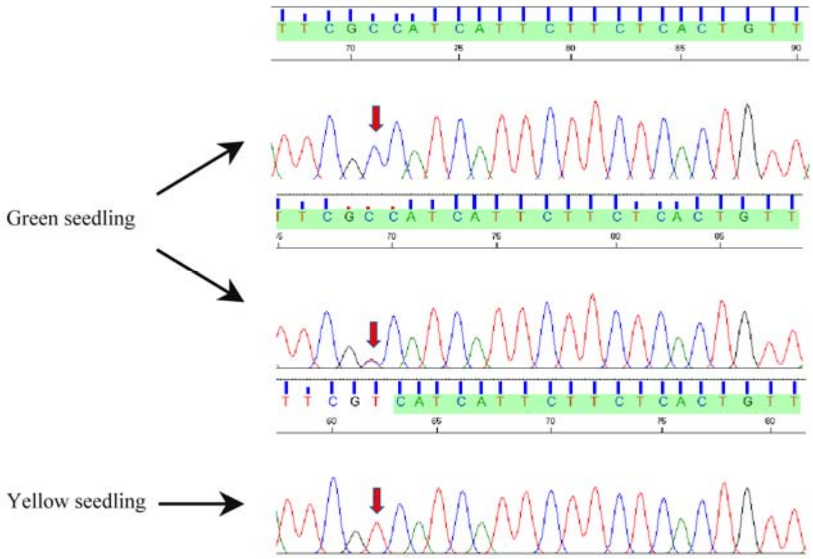


Figure S3 Genotype of the causal mutation in plants with different phenotype. Red arrow indicates the 2771134$^{th}$ of chromosome 1.

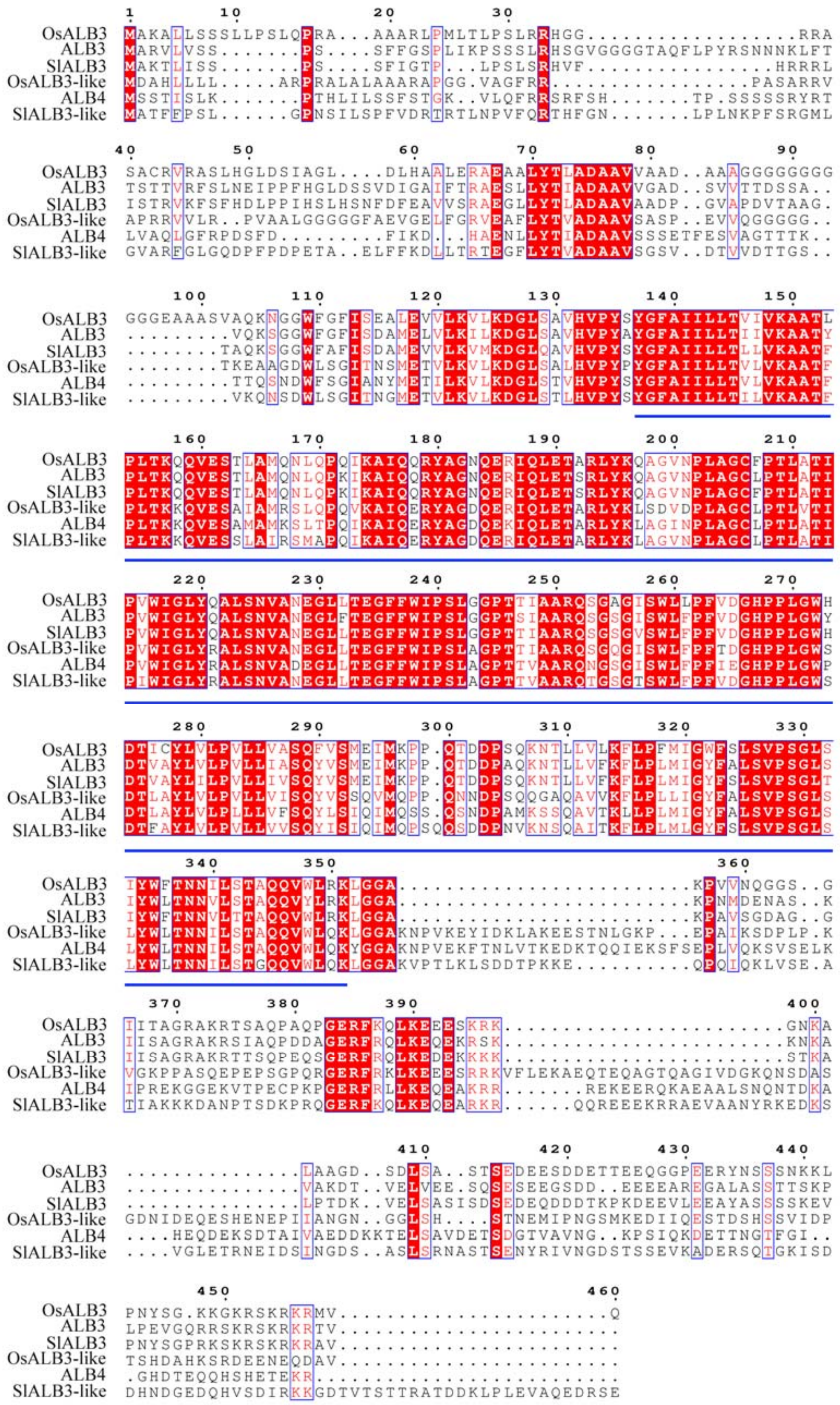

Figure S4 Multiple sequence alignment of the ALB3 and ALB3-like proteins in *Oryza sativa*, *Arabidopsis thaliana*, and *Solanum Lycopersicum*. The conserved YidC_Oxa1_Cterm domain is underlined in blue.

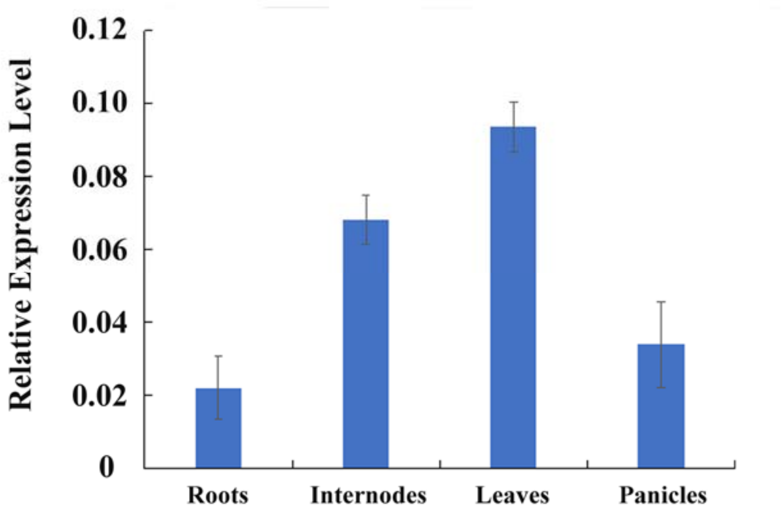


Figure S5 Real-time PCR analysis of *OsALB3* in different rice tissues. *Ubiquitin* is used as endogenous control. Error bars represent SD (n = 3)

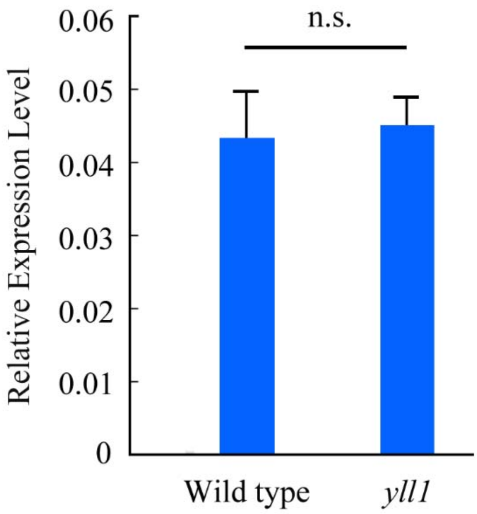


Figure S6 Comparison of expression level of *OsALB3* between wild type and *yll1*
Seedling leaves were used for real-time PCR analysis. *Ubiquitin* is used as endogenous control. Error bars represent SD (n = 3). n.s. indicate no significant differences according to two-tailed Student's *t* test.

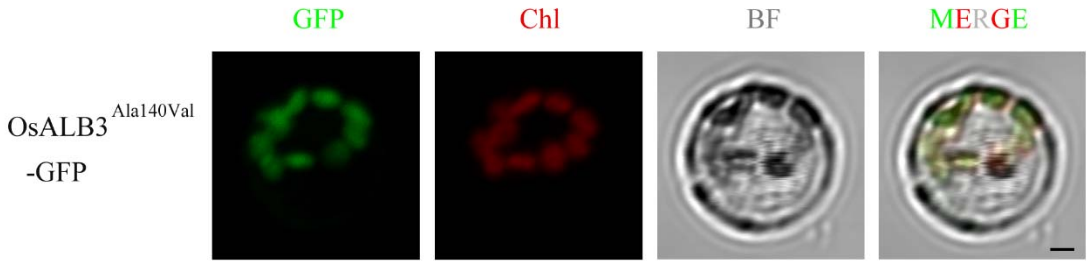


Figure S7 Subcellular localization of OsALB3$^{Ala140Val}$ in rice protoplasts

Green fluorescence indicates GFP signal, red fluorescence indicates chloroplast autofluorescence.

Chl, chloroplast. BF, bright field. Scale bar=5 μm.

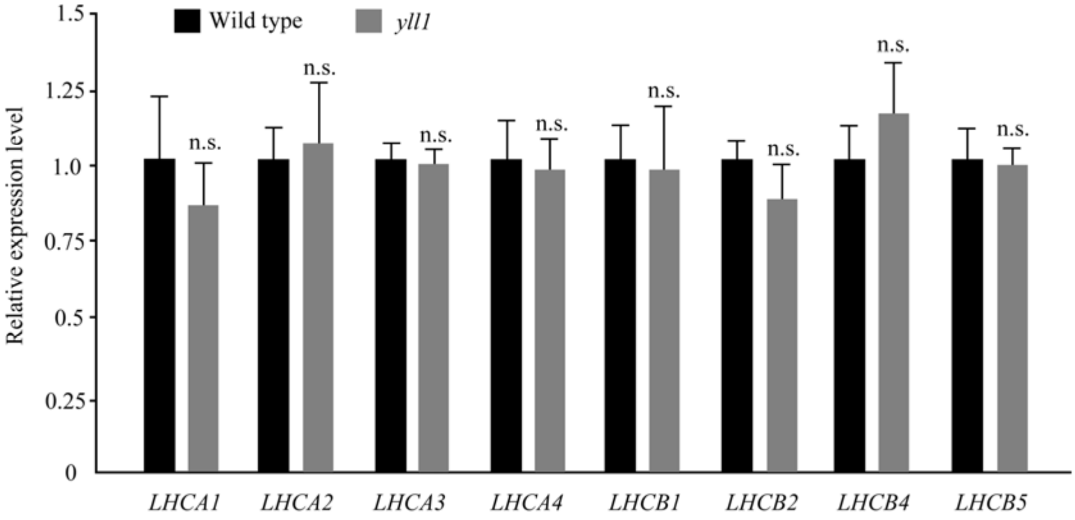


Figure S8 Comparison of expression level of LHCPs encoding genes between wild type and *yll1* Seedling leaves were used for real-time PCR analysis. *Ubiquitin* is used as endogenous control. Error bars represent SD (n = 3). n.s. indicate no significant differences according to two-tailed Student's *t* test.
